# Surgical Management and Prognosis of Congenital Choledochal Cysts in Adults: A Single Asian Center Cohort of 69 Cases

**DOI:** 10.1155/2022/9930710

**Published:** 2022-01-21

**Authors:** Jianchun Xiao, Minting Chen, Tao Hong, Qiang Qu, Binglu Li, Wei Liu, Xiaodong He

**Affiliations:** ^1^Department of General Surgery, Peking Union Medical College Hospital, Chinese Academy of Medical Sciences & Peking Union Medical College, No. 1 Shuaifuyuan, Beijing 100730, China; ^2^Chinese Academy of Medical Sciences & Peking Union Medical College, Beijing 100005, China

## Abstract

**Background:**

The choledochal cyst (CC) is a rare cystic dilatory condition with malignant tendency, which is more frequently reported in children. Surgical resection of cysts can significantly decrease the risk of malignancy and reduce associated complications. However, CC has been paid lesser attention in adults, and its surgical parameters have been frequently reported to be in dispute. This study aimed to report experience associated with the treatment of an adult with CC and to suggest the appropriate parameters for the surgery, including the extent of excision (complete or not), the length of the Y limb, the diameter of the cholangio-intestinal anastomosis (CIA), and different operative approaches (open, laparoscopic, and laparoscopic converted to open) by comparing the various indicators, including postoperative bile leakage, cholangitis, choledocholithiasis, carcinogenesis, and surgical re-excision.

**Methods:**

We conducted a single-center noninterventional retrospective study of 69 different congenital choledochal cyst patients who were admitted to our hospital between July 2010 and July 2020. We collected and analyzed their demographic data, clinical presentations, underlying complications, imaging tests, endoscopic interventions, and parameters for the surgery, histological data, and prognostic indicators over a mean 77-month follow-up period.

**Results:**

We found that out of the 69 cases, the median age at diagnosis was 32 (IQR = 22–45) years. Seven (10.1%) patients were asymptomatic before the diagnosis, with abdominal pain as the primary complaint in 62 (89.9%) patients, whereas nausea/vomiting was observed in 29 (42.0%) patients. CCs were mainly evaluated by using magnetic resonance cholangiopancreatography (MRCP) (*n* = 47, 68.1%). It was observed that surgery, cholecystectomy, choledochal cysts excision, and Roux-en-Y hepaticojejunostomy (*n* = 65, 94.2%), and laparotomy (*n* = 58, 84.1%) were the dominant therapeutic modalities employed. However, seventeen (24.6%) patients were treated with incomplete cyst resection, while 52 (75.4%) patients received complete cyst resection. We also conducted regular follow-ups after the surgery for a mean duration of 77 months. Postoperative complications were found to be experienced by 35 (50.7%) patients, and a further two patients (2.9%) developed malignancy during the follow-up. Moreover, increasing the diameter of cholangio-intestinal anastomosis served as a potential protective factor for postoperative choledocholithiasis (*p* = 0.040) and a risk factor for cholangitis (*p* = 0.002).

**Conclusions:**

Among the 69 CC participants, abdominal pain was their major symptom. Those with an abnormal pancreaticobiliary junction were more likely to have choledocholithiasis and pancreatitis. The diagnosis was found to be highly dependent on the Todani classification scheme and MRCP. Surgical resection remains the key to CC treatment. The results suggested that the complete resection, the length of the Y limb of 40 cm–60 cm, and the diameter of the CIA of 1.0 cm–1.5 cm were appropriate values for predicting the lower risk of postoperative complications.

## 1. Introduction

Congenital choledochal cyst (CC) is a relatively rare inborn bile duct abnormality that is predominantly characterized by cystic dilation of the biliary tract that is usually accompanied by an abnormal pancreaticobiliary junction (APBJ) [1]. There are five different types of CC as described in the Todani classification system [2], among which type I and IV cysts have been found to be more prevalent in Asian populations and more frequently associated with the presence of APBJ [3]. The classical presentations of this disease include nausea/vomiting and other important elements of the triad of fever, abdominal pain, and jaundice. Besides, CC often coexists with various complications such as cholecystitis, cholangitis, pancreatitis, stone formation, and malignancy. Overall, the reported prevalence of cancer has been found to be variable, and between 3% and 30% of CC patients have been reported to develop cancer [4, 5]. The cancer risk increases significantly with age and has been found to be higher in those with type I and IV cysts. A number of previously reported studies on CC have been predominantly based on children because the majority of patients who developed symptoms were found to be under the age of 10 years [6]. Moreover, total cyst resection has been advocated for management [7] because subsequent biliary cancer and postoperative complications such as lithiasis could be prone to develop in the cyst remnant [8, 9]. However, it might not be technically feasible to completely remove the cysts because of their intrahepatic or intrapancreatic nature [10]. In this case, some surgeons prefer to conduct only partial hepatectomy [11] or intramural dissection of the intrapancreatic biliary cyst [12] to achieve complete excision. However, an opposite opinion has been proposed that clinical outcomes in patients who have only received partial excisions can remain optimal as long as the proper bile flow has been established [13]. Besides, the poor prognosis of CC such as ascending inflammation and anastomotic stricture has also been reported to be associated with an inappropriate length of Y limb or diameter of cholangio-intestinal anastomosis [14]. Therefore, we have focused on the surgical management and clinical outcome of choledochal cysts in Asian grown-ups with an aim to provide an experience reference for the standardization of treatment modalities for adult patients with CC.

## 2. Methods

All patients who had received surgery for any type of CC at our hospital from July 2010 to July 2020 were enrolled in this study. Surgical indications depended on whether the patient displayed symptoms and the type of cyst detected, given their various surgical difficulties and malignant potential [15]. The type of cyst present was preliminarily defined by the Todani classification system and determined by the various imaging methods, including B-ultrasound, magnetic resonance cholangiopancreatography (MRCP), endoscopic retrograde cholangiopancreatography (ERCP), or abdominal computed tomography. We recommended surgery for patients with type I, II, and IV cysts and symptomatic or asymptomatic young patients with type III cysts. Patients with type V cysts generally receive supportive treatment rather than surgery, aiming to control the various complications such as recurrent cholangitis and sepsis, but some patients eventually need liver transplantation [16]. Moreover, their preoperative imaging, intraoperative anatomy, and postoperative pathology all confirmed the diagnosis of bile duct cyst. A written/verbal informed consent was provided by each participant, and the study was approved by the Institutional Review Board of our hospital (approval number S-K1609).

The demographic and clinical data were thereafter collected, including information about the gender, age at diagnosis, the type of cyst detected, associated symptoms, complications, results of imaging tests and endoscopic interventions, operative approaches (open or laparoscopic), the extent of excision, loss of blood, length of Y intestinal limb, the diameter of cholangio-intestinal anastomosis (CIA), pathological findings, and the length of hospital stay. The patients were interviewed regularly to obtain the necessary information about the postoperative complications (especially malignant transformation) and the secondary operation updates, with the most recent follow-up conducted in January 2021.

All data were analyzed using IBM SPSS Statistics for Windows, version 25.0 (IBM Corp., Armonk, NY, USA). Categorical variables were presented in numbers (%) or (numbers, %) and assessed using the Pearson *χ*2 test or Fisher's exact test wherever appropriate. Continuous variables were depicted as medians with interquartile range (IQR) or mean ± standard deviation and analyzed using the Wilcoxon rank-sum test or independent sample *t*-test wherever appropriate. A two-sided *p*value <0.05 was considered statistically significant.

## 3. Results

It was found that out of the 69 cases analyzed, 18 (26.1%) were men and 51 (73.9%) were women. The demographic and clinical characteristics of these study participants have been presented in Table 1. The median age at the diagnosis was 32 (IQR = 22–45) years. The time interval from discovery to the first surgical treatment was 72 ± 14 months. The mean hospital stay was 18 ± 1 days. The mean duration of the follow-up was 77 ± 8 months. The majority of these 69 patients were symptomatic, with the most common complaints reported being those of abdominal pain (62, 89.9%), nausea/vomiting (29, 42.0%), and fever (21, 30.4%). CC was an incidental finding in 7 (10.1%) cases. The various observed complications included cholecystitis (69, 100%), choledocholithiasis (32, 46.4%), cholecystolithiasis (27, 39.1%), cholangitis (26, 37.7%), pancreatitis (15, 21.7%), and biliary tumor (5, 7.2%). Among the 44 (63.8%) patients with CC and APBJ, 29 (65.9%) were found to be type I, and 13 (29.5%) were type IV. There was no significance in demographic characteristics, presentations, APBJ, malignancy, and other complications between the patients with type I CC and type IV CC. Among the 69 CC participants, the patients with APBJ were more likely to have choledocholithiasis (*p* = 0.001) and pancreatitis (*p* = 0.007) (Table 2).

**Figure 1:**
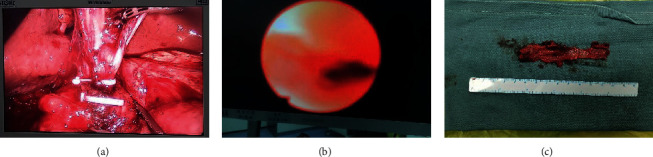
Representative images of a congenital choledochal cyst. (a) Laparoscopic view. (b) Endoscopic view. (c) Specimen.

Moreover, a variety of supplementary modalities (Figure 1) were used for diagnosis and treatment, including magnetic resonance cholangiopancreatography (MRCP) (47, 68.1%), endoscopic retrograde cholangiopancreatography (ERCP) (13, 18.8%), percutaneous transhepatic cholangial drainage (PTCD) (4, 5.8%), and endoscopic nasobiliary drainage (ENBD) (2, 2.9%) as shown in Table 3. Moreover, by taking the Todani classification system as the imaging diagnostic criteria, the different types of the choledochal cyst were defined as I (47, 68.1%), II (1, 1.4%), III (1, 1.4%), IV (19, 27.5%), and V (1, 1.4%).

During the first surgery to remove the choledochal cyst (Table 4), 65 (94.2%) patients underwent cholecystectomy, choledochal cyst excision, and Roux-en-Y hepaticojejunostomy, whereas 2 (2.9%) patients underwent partial hepatectomy additionally. For the Roux-en-Y hepaticojejunostomy, the diameter of the CIA ranged from 0.5 cm to 3.0 cm with an average of 1.3 ± 0.1 cm, and the length of the Y-intestinal limb was found to lie between 40 cm and 60 cm with an average of 49 ± 1 cm. 3 (4.6%) patients had radical resection of cholangiocarcinoma. The only patient (1.5%) detected with type III cyst underwent open cholecystectomy, choledochal cyst excision, and duodenal papillary reconstruction, while the patient (1.5%) diagnosed with type V cyst received laparoscopic fenestration drainage in the hepatic cysts. The surgical approach was laparoscopic (8, 11.6%), open (58, 84.1%), and laparoscopic converted to open (3, 4.3%) for better management. Moreover, seventeen (24.6%) patients were treated with incomplete cyst resection, while 52 (75.4%) patients received complete cyst resection.

Interestingly, the pathological findings of all the resected specimens clearly verified the diagnosis of CC. These CCs were mainly localized in the normal bile duct mucosa. Some CCs displayed an inflammatory profile with erosion and sparse mucinous glands, whereas some others showed metaplasia and biliary intraepithelial neoplasia. Among them, there were 5 participants who showed concomitant malignancy, including 3 (60%) cholangiocarcinoma (CCA) and 2 (40%) gallbladder adenocarcinoma (Table 5). Notably, these five patients were all female. The average age at which they were diagnosed with cancer was 48 years. Moreover, compared with the whole cohort, these cancer patients were diagnosed with CC at a later age, with an average of 45 years. The course of the disease in these patients was also significantly shorter, with an average of 38 months. All of the five patients underwent cholecystectomy, choledochal cyst excision, and Roux-en-Y hepaticojejunostomy during the first surgery, with an average Y-limb length of 50 cm and an average anastomotic diameter of 2.1 cm. However, three of them underwent open surgery, one received laparoscopy, and one was converted to open surgery. Additionally, two cases of type I CC were subjected to complete resection. Of the three patients with type IV CC, one had an incomplete resection whereas the other two underwent total removal of the cystic tissues including one who had a partial hepatectomy. Similar to the whole cohort, the average length of hospital stay was 17 days. The average blood loss was 246 ml, which was significantly more than that of the whole cohort. After an average follow-up of 56 months, four of the five patients developed cholangitis and one had choledocholithiasis, but all of them were still alive.

During the first surgery, the average blood loss was 144 ± 19 ml. However, by the last follow-up in July 2021, 34 (49.3%) patients experienced no major complications after the surgical treatment (Table 6). Additionally, the different postoperative manifestations included bile leakage in 3 (4.3%), choledocholithiasis in 14 (20.3%), cholangitis in 30 (43.5%), and the malignant transformation in 2 (2.9%). 12 (17.4%) patients received a second surgery, and the observed results clearly indicated that patients who had CC remnants were more likely to receive surgical re-excision (*p* = 0.025). There was no significant difference in the amount of bleeding between the patients who received incomplete surgery and patients who underwent complete surgery, who neither had any other postoperative outcomes nor did they have any difference in the length of hospital stay.

Thereafter, statistical analysis (Table 7) was applied to explore the potential correlation between postoperative outcomes (greater volume of blood loss during surgery, longer length of hospital stay, malignant transformation, bile leakage, choledocholithiasis, cholangitis, and surgical re-excision) as well as the potential risk factors (age at diagnosis, gender, type of CC, length of the Y limb, the diameter of cholangio-intestinal anastomosis, and operative approaches). It was indicated that increasing the diameter of the cholangio-intestinal anastomosis could serve as a protective factor for postoperative choledocholithiasis (*p* = 0.040) and a risk factor for cholangitis (*p* = 0.002). Laparoscopic surgery was associated with an increased risk of bile leakage (*p* = 0.010). Age at diagnosis was found to be a risk factor for carcinogenesis (*p* = 0.002) and bile leakage (*p* = 0.002). Among the three (4.3%) patients with postoperative biliary leakage, two (2.5%) cases underwent laparoscopy and one (1.7%) patient underwent a laparotomy.

## 4. Discussion

Congenital choledochal cysts are cystic dilations with a reported incidence of about 1 : 1,000 in Asian populations [17]. It has been found that women are more likely to have CC, with a female-to-male ratio of about 3 : 1 [18], which was confirmed by our study. According to the number and position of the cysts and the presence of APBJ, the Todani system has elegantly categorized CC into five different types. Type I refers to the cystic or fusiform dilations of the common bile duct except for the intrahepatic component. Morphologically, they can be further categorized into three distinct subtypes, namely type IA, type IB, and type IC. On the contrary, type II is a diverticular dilatation of the extrahepatic bile duct, whereas type III is a choledochocele limited to the duodenal wall of the distal common bile duct. Type IV depicts multiple dilations with two different subtypes. Type IVA shows both intrahepatic and extrahepatic bile ducts, while type IVB has been found to be only limited to extrahepatic portions. Type V is cystic dilation confined only to the intrahepatic ducts. Among all the different types of reported CCs, type IA, IC, and IVA are often present with APBJ and are more frequently diagnosed [2]. Type I and IV were more commonly seen in the 69 patients, which accords to the formal studies, with 50–85% of the reported cysts being categorized as type I which was found to be followed by type IV at 15–35% [2, 19].

APBJ can occur in 50%–80% of patients with CC [20], as verified by this study. It is a congenital malformation primarily characterized by an early pancreaticobiliary union before entering into the duodenal wall. The major diagnostic criteria of ABPJ are that the common ductal channel is longer than 8 mm and the bile amylase concentration is higher than 8000 UI/L. It has been found that APBJ allows constant two-way pancreaticobiliary regurgitation, which can ultimately result in the chemical exchange, chronic biliary/pancreatic inflammation, cyst formation, presence of protein thrombi or stones in the common channel, and finally can significantly increase the risk of the subsequent carcinogenesis [21]. We observed that 61.4% of our participants with APBJ were accompanied by choledocholithiasis and 31.8% were diagnosed with pancreatitis, thus supporting the causative role of APBJ.

It has been reported that children are a high-risk population of CC and can often manifest some or all of the triad of abdominal pain, jaundice, and abdominal mass. On the contrary, the majority of adult CC patients commonly present with fever, nausea/vomiting, as well as pain and jaundice [22]. The overall symptoms and nonmalignant complications found in analyzed patients appeared to be similar to those of previous study cohorts [23], with abdominal pain and cholecystitis being observed to be the most common symptoms and complications, respectively.

Imaging modalities play a pivotal role in the detection, diagnosis, classification, and supplement treatment for CC. It has been found that MRCP has a sensitivity of 73–100% for diagnosing CC, which is markedly less than that of direct cholangiography such as ERCP [24]. However, MRCP remains the first choice in most clinical centers, including ours, predominantly to avoid radiation exposure, cholangitis, and pancreatitis associated with invasive cholangiography.

Surgical removal of the CC remains the treatment of choice once the diagnosis of type I, II, or IV has been confirmed, while therapy for type III and V CC would often be less aggressive comparatively [25]. All CC patients that were admitted to our hospital underwent prophylactic cholecystectomy to prevent carcinogenesis [26]. Complete resection has been found to serve as a potentially protective factor against the various postoperative nonmalignant complications such as cholangitis, choledocholithiasis, pancreatitis, and carcinogenesis in several of the previous study cohorts [27]. However, complete resection of some intrahepatic and intrapancreatic cysts might not be technically feasible. Besides, dissection of the intrahepatic cysts may effectively cause anastomotic stricture with resultant cholangitis and choledocholithiasis due to the relatively small diameter of the secondary bile duct. In addition, the removal of intrapancreatic cysts can significantly increase the risk of pancreatic duct injury. Hence, some surgeons could also consider partial hepatectomy and pancreatoduodenectomy to achieve a complete resection [28]. However, if the operator or patient does not agree to sacrifice the normal liver or pancreas, or is reluctant to bear the risk of different associated complications, partial resection can only serve as a second choice. It was observed in one previous reported Asian study that cancerous and precancerous lesions were more frequently identified in type IV patients who underwent additional hepatectomy than those who did not [7]. Besides, Xia et al. also demonstrated that establishing proper bile flow was more significant than total excision for achieving good clinical outcomes, thereby suggesting that partial excision could be accepted as long as the problem of pancreaticobiliary regurgitation can be resolved [13]. In our study, complete excision was only significantly associated with, yet complete excision of intrapancreatic cysts of type I and the intrahepatic portion of type IV showed no significant association with better postoperative outcomes, respectively. Nevertheless, we recommend an aggressive surgical procedure to remove all of the cyst tissue with the best effort. In conclusion, the extent of excision still remains controversial. In our study, complete excision showed a significant association with a lower surgical re-excision rate only. Nevertheless, we recommend aggressive surgical procedures to remove all of the cyst tissues with the best effort. This is primarily because the anatomy of reoperation has been found to be particularly difficult due to the serious adhesion of the abdominal structure after the first operation.

Laparoscopic surgery has been gradually accepted and commonly used in recent decades for the management of CC. It requires a learning curve to master the various laparoscopic skills because the needle entry angle, suture accuracy, anastomotic tension, and other factors have been found to be significantly different between open surgery and one performed under laparoscopic conditions. However, laparoscopy offers the advantages of a smaller wound (especially for young women who care particularly about appearance and account for major proportions of patients), faster recovery, lower probability of intestinal adhesion or obstruction, and longer survival [29]. Therefore, it is necessary to constantly practice laparoscopic skills with the increasing demand for laparoscopic surgery.

Moreover, Roux-en-Y hepaticojejunostomy was also performed where the jejunum was cut off and the distal segment (the Y limb) was raised to anastomose with the bile duct, and thereafter the proximal intestinal segment was anastomosed with the distal jejunum. The Y-limb constitutes the lifted segment of the intestine, which is mainly used to shunt bile and intestinal contents, while its absorption function has been seriously hampered. The maintenance of the proper length of the Y limb remains crucial. We suggest based on our findings that the optimal length of the Y intestinal limb should be less than 60 cm. If the Y limb is too long, it might be easy to twist, fold, or droop, which can significantly affect the absorption function of the small intestine. In this case, the patients may suffer from malnutrition and bacterial overgrowth in the small intestine [30]. On the contrary, it is generally believed that the antireflux ability could be positively correlated with the length of the Y limb, and the Y limb should be longer than 40 cm, as we have observed in clinical practice. However, the length of children's Y limb is often limited because they need enough functional intestine to meet their nutritional needs of the growth, and therefore, they are likely to have the intestinal content reflex and associated ascending cholangitis, choledocholithiasis, and anastomotic stricture due to the relatively shorter length of their Y limb [14]. Alternative surgical options that can be implemented for the children include jejunoduodenostomy with interposition of a bile duct and choledochoduodenostomy. The patient, who had developed symptoms since the age of 3, also underwent choledochoduodenostomy in his childhood and died of malignant transformation (Table 5). In conclusion, we recommend an optimal length of 40 cm–60 cm of intestinal Y limb for adult CC patients.

Besides, another key point of Roux-en-Y hepaticojejunostomy was to avoid an anastomotic stricture of the bile duct as well as the jejunum and to maintain them in an unobstructed state. The anastomotic stoma tends to display stenosis because the common bile duct is unable to effectively contract and expand by itself due to the lack of well-developed smooth muscles in the intrahepatic and extrahepatic bile ducts, especially in the case of secondary chronic cholangitis that can therefore lead to significant fibrosis and thickening of the bile duct wall. Therefore, the anastomosis should be as large as possible, and the mucosa of the bile duct should be anastomosed with the mucosa of the small intestine [30]. During our surgical procedures, the sites where the thickness of the bile duct changed significantly were taken as the cutting edge. We concluded that 1cm–1.5 cm could serve as a proper range for the diameter of CIA because the patients with a larger diameter of anastomosis were substantially more likely to develop choledocholithiasis and those with the smaller diameters tended to suffer from cholangitis. Moreover, an inappropriate length of Y limb and the diameter of cholangio-intestinal anastomosis (CIA) have been found to be closely related to reflux cholangitis or bile duct stenosis. Repeated inflammation can significantly increase the risk of cancer. Therefore, we did statistical analysis including the two different factors as independent variables and postoperative carcinogenesis as dependent variables but failed to get proper statistical significance. This finding clearly indicated that there was no direct association between the length of the Y limb and cancer development, nor did the diameter of the CIA based on current cases.

CC patients have been found prone to develop malignant tumors, such as cholangiocarcinoma, gallbladder adenocarcinoma, and pancreatic cancer, whose prognoses are very bad. The risk of malignant transformation has been suggested to be associated with cyst type, age, and the presence of APBJ [7]. However, we managed to prove part of the conclusion only, given that the sample size of postoperative malignant transformation was too small. Except for one patient who was 23 years old, all the others were over 40 years old when they were diagnosed with cancer, which accords to the age-adjusted strategy proposed by Voyles et al. [31] and proves the correlation between age and malignancy.

The two patients who developed postoperative cancer both had type IV cysts and died shortly after the discovery of malignant transformation. The first patient was a 52-year-old woman who presented with a fever, jaundice, abdominal pain, and nausea. She also suffered from complications that included cholecystolithiasis, choledocholithiasis, cholecystitis, and cholangitis. After examination with ERCP and MRCP, she underwent cholecystectomy, choledochal cyst excision, and Roux-en-Y hepaticojejunostomy with the Y limb length of 50 cm and a diameter of CIA of 3.0 cm. The cyst was confirmed to be type IV during operation, and no cancer tissue was found by pathology. A few days later, the patient was discharged from our hospital as her general condition was satisfactory. However, in the following six months, the patient's symptoms recurred and deteriorated rapidly, and positron emission tomography CT showed multiple high glucose uptake signals in the lung and peritoneum, suggesting that the patient had a malignant transformation and lost the operation indication. After several months of conservative treatment, the patient died of an electrolyte disorder. The time from the operation to the detection of cancer was very short, which may be due to the rapid process of malignant transformation. However, it is also possible that the intrahepatic segment cyst had malignancy originally. We failed to detect malignant tissue as early as possible because of the limitations of imaging methods, and the intrahepatic bile ducts were too thin to be biopsied during the operation. The second patient was diagnosed to have type IV cysts at the age of 3 with symptoms including fever, jaundice, and abdominal pain and complications including choledocholithiasis, cholecystitis, and cholangitis. He underwent choledochocyst duodenostomy because of his young age. In the following 40 years, he suffered from recurrent choledocholithiasis and cholangitis, which finally drove him to our hospital for a second surgery. By then, his abdominal cavity was full of inflammatory hyperplasia, serious adhesion, and hardened connective tissue which made anatomical marks unclear and hard to operate. Based on the intraoperative conditions and postoperative paraffin pathology, the patient was finally diagnosed as T3N1M0, a moderately poorly differentiated cholangiocarcinoma. The patient was unable to survive due to serious cachexia and died after an ineffective rescue during hospitalization. These two cases suggest that incomplete surgery may leave residual malignant tissue, which is difficult to deal with once found because of the impact of the first operation. Moreover, the cancer tissue may have metastasized, resulting in the loss of intervention opportunities.

Regular follow-up is of great significance in both the prevention and treatment of postoperative complications. In our experience, bile leakage, cholangitis, and choledocholithiasis were the most common posttreatment complications found.

There are several limitations associated with our study, the majority of which are hospital-based and retrospective in design. As one of the most comprehensive tertiary hospitals, our hospital mainly accepts severe and difficult surgical cases that could not be handled effectively by other clinical centers which may cause some bias. Secondly, we failed to report the data related to the clinical presentation, surgical management, and prognosis of those CC patients who did not meet the surgical indications and were thus excluded from our study cohort. Thirdly, the sample size of 69 is relatively small, and not all cyst subtypes were more than 10, and there were also only 2 cases of postoperative malignant transformation. Finally, we could not correlate most of the observed postoperative complications with the specific potential risk factors.

Most of the previously reported cases were children, given the early onset of CC, but adult patients are gradually getting attention. We collected and analyzed the demographic and clinical data of all adult CC patients who underwent surgery in our hospital from 2010 to 2020. Adults tend to have negative signs of abdominal mass on physical examination, but they are more likely to have nausea and vomiting. MRCP is a common preoperative examination method for both children and adults. We recommend cholecystectomy, CC resection, and Roux-en-Y hepaticojejunostomy for our adult patients. Considering the pathology and prognosis of patients, we support the total resection scheme, which reduces the probability of difficult reoperation and avoids missing cancer tissue in cyst remnants. Adult intestines are long enough to accept the appropriate length of Y-loop, ranging from 40 cm to 60 cm. Besides, the biliary-enteric anastomotic stoma should be as large as possible, as long as the CC is resected completely. This is of great significance to ensure the proper diversion of bile and food, thereby reducing inflammation, stones, and anastomotic stenosis. Finally, surgeons should improve surgical skills to meet the growing demand for laparoscopy.

## 5. Conclusion

As a serious premalignant condition, a congenital choledochal cyst should be carefully removed surgically and kept under regular surveillance. The recommended procedures include laparoscopic cholecystectomy, complete cyst excision, and Roux-en-Y hepaticojejunostomy with an optimal length of intestinal Y limb of 40 cm–60 cm and a diameter of cholangio-intestinal anastomosis of 1 cm–1.5 cm.

## Figures and Tables

**Table 1:** Demographic and clinical characteristics of the study participants.

Characteristics	Overall	Type I	Type IV	*p* value
Gender, *n* (%)	69 (100)	47 (68.1)	19 (27.5)	0.947
Female	51 (73.9)	35 (68.6)	14 (27.4)	-
Male	18 (26.1)	12 (66.7)	5 (27.8)	-
Age at diagnosis (years)	32 (22–45)	33 ± 3	35 (27–41)	0.202
Disease course (months)	72 ± 14	66 ± 15	93 ± 35	0.082
Length of hospital stay (days)	18 ± 1	18 ± 2	18 ± 2	0.397
Follow-up (months)	77 ± 8	83 ± 11	69 ± 12	0.677

Presentation, *n* (%)				
Abdominal pain	62 (89.9)	43 (69.4)	17 (27.4)	1.000
Abdominal mass	3 (4.3)	1 (33.3)	2 (66.7)	0.197
Jaundice	18 (26.1)	13 (72.2)	4 (22.2)	0.759
Fever	21 (30.4)	15 (71.4)	5 (23.8)	0.654
Charcot's triad	12 (17.4)	10 (83.3)	1 (8.3)	0.156
Nausea/vomit	29 (42.0)	18 (62.1)	10 (34.5)	0.286
Incidental finding	7 (10.1)	4 (57.1)	2 (28.6)	1.000
APBJ	44 (63.8)	29 (65.9)	13 (29.5)	0.607

Complication, *n* (%)	69 (100)			
Malignancy	5 (7.2)	2 (40.0)	3 (60.0)	0.139
Cholecystolithiasis	27 (39.1)	17 (63.0)	10 (37.0)	0.218
Choledocholithiasis	32 (46.4)	22 (68.8)	9 (28.1)	0.967
Cholecystitis	69 (100)	47 (68.1)	19 (27.5)	-
Cholangitis	26 (37.7)	16 (61.5)	9 (34.6)	0.312
Pancreatitis	15 (21.7)	9 (60.0)	5 (33.3)	0.519

APBJ: abnormal pancreaticobiliary junction.

**Table 2:** Correlation of the various observed complications with an abnormal pancreaticobiliary junction (APBJ).

APBJ	N (%)	*p* value
Malignancy	3 (6.8)	1.000
Cholecystolithiasis	17 (38.6)	0.911
Choledocholithiasis	27 (61.4)	0.001
Cholecystitis	44 (100)	—
Cholangitis	18 (40.9)	0.284
Pancreatitis	14 (31.8)	0.007

APBJ: abnormal pancreaticobiliary junction.

**Table 3:** Supplementary modalities for the diagnosis and treatment.

Operation	Overall, *n* (%)	Type I, *n* (%)	Type II, *n* (%)	Type III, *n* (%)	Type IV, *n* (%)	Type V, *n* (%)
ERCP	13 (18.8)	10 (76.9)	0	0	3 (23.1)	0
PTCD	4 (5.8)	3 (75.0)	0	0	1 (25.0)	0
MRCP	47 (68.1)	33 (70.2)	1 (2.1)	1 (2.1)	12 (25.5)	0
ENBD	2 (2.9)	2 (100)	0	0	0 (0)	0
Overall	69 (100)	47 (68.1)	1 (1.4)	1 (1.4)	19 (27.5)	1 (1.4)

MRCP: magnetic resonance cholangiopancreatography, ERCP: endoscopic retrograde cholangiopancreatography, PTCD: percutaneous transhepatic cholangial drainage, and ENBD: endoscopic nasobiliary drainage.

**Table 4:** Choledochal cyst types and the first surgical procedures undertaken.

Cyst type	I	II	III	IV	V	Overall
Laparoscopy, N (%)	6 (12.8)	—	—	1 (5.3)	1 (100)	8 (11.6)
Laparotomy, N (%)	38 (80.9)	1 (100)	1 (100)	18 (94.7)	—	58 (84.1)
LS conversed to LT, N (%)	3 (6.4)	—	—	0 (0)	—	3 (4.3)
Length of Y (cm)	48 ± 1	55	—	51 ± 1	—	49 ± 1
Diameter of the CIA (cm)	1.2 ± 0.1	2.5	—	1.5 ± 0.2	—	1.3 ± 0.1
CC and RYHJ	47 (100)	1 (100)	—	16 (88.9)	—	65(94.2)
CC, RYHJ, and PH	—	—	—	2 (11.1)	—	2 (2.9)
CC and DPR	—	—	1 (100)	—	—	1 (1.5)
FDHC	—	—	—	—	1 (100)	1 (1.5)
RRC	1 (2.4)	—	—	2 (11.1)	—	3 (4.6)
Complete resection	43 (91.5)	1 (100)	1 (100)	6 (31.6)	1 (100)	52 (75.4)
Incomplete resection	4 (8.5)	—	—	13 (68.4)	—	17 (24.6)

LS: laparoscopy, LT: laparotomy, CC: cholecystectomy and choledochal cysts excision, RYHJ: Roux-en-Y hepaticojejunostomy, PH: partial hepatectomy, DPR: duodenal papillary reconstruction, FDHC: fenestration drainage in hepatic cysts, and RRC: radical resection of cholangiocarcinoma.

**Table 5:** Demographic and clinical data of patients with concomitant malignancy or developed postoperative malignancy.

AD/sex	DC (m)	Follow-up (m)	AM	Cyst type	Symptoms	APBJ and complications	First surgery	CE	Length of the Y limb (cm)	Diameter of the CIA	Surgical approach	Other operations	HS	BL	*P*C	Pathology	SR	Outcome
36/F	96	47	44	IV	Abdominal pain, nausea/vomit	APBJ, malignancy, cholecystitis	CCE and RYHJ	—	—	—	1	no	20	—	Cholangitis	Moderately differentiated CCA, T2N0M0	Yes	Alive
23/F	2	37	23	IV	Abdominal pain, jaundice, nausea/vomit	APBJ, malignancy, cholecystolithiasis, choledocholithiasis, cholecystitis, cholangitis	RRC and PH	Yes	50	2.0	1	MRCP	19	300	Cholangitis	Well-differentiated CCA, T2N0M0	No	Alive
65/F	1	65	65	I	Incidental finding	APBJ, malignancy, cholecystitis	CCE, RYHJ, and RRC	Yes	50	1.2	3	no	18	400	no	Well-differentiated CCA, T1N0M0	No	Alive
63/F	8	32	63	I	Charcot's triad, nausea/vomit	Malignancy, cholecystolithiasis, cholecystitis, pancreatitis	CCE and RYHJ	Yes	50	—	2	No	15	80	Cholangitis	Well-differentiated GAC, T2N0M0	No	Alive
39/F	84	100	46	IV	Abdominal pain	Malignancy, cholecystolithiasis, cholecystitis	CCE, RYHJ, and RRC	No	50	2.0	1	MRCP	13	150	—	GAC, T3N0M0	-	Alive
52/F	1	124	53	IV	Charcot's triad, nausea/vomit	APBJ, cholecystolithiasis, choledocholithiasis, cholecystitis, cholangitis	CCE and RYHJ	No	50	3.0	1	ERCP, MRCP	23	300	Malignancy	T4N2M1	Yes	Deceased
3/M	480	440	43	I	Charcot's triad	APBJ, choledocholithiasis, cholecystitis, cholangitis	Choledochocyst duodenostomy	-	45	—	1	—	73	-	Malignancy, choledocholithiasis, cholangitis	Moderately poorly differentiated CCA, T3N1M0	Yes	Deceased

AD: age at diagnosis (years), F: female, M: male, DC: disease course (months), AM: age when malignancy discovered (years), CE: complete excision, surgical approaches include 1, open; 2, laparoscopic; and 3, laparoscopic converted to open, HS: hospital stay (days), BL: blood loss (mL), PC: postoperative complication, SR: surgical re-excision, CCE: cholecystectomy and choledochal cysts excision, CIA: cholangio-intestinal anastomosis, ERCP: endoscopic retrograde cholangiopancreatography, MRCP: magnetic resonance cholangiopancreatography, CCA: cholangiocarcinoma, GAC: gallbladder adenocarcinoma, PH: partial hepatectomy, RRC: radical resection of cholangiocarcinoma, and RYHJ: Roux-en-Y hepaticojejunostomy.

**Table 6:** Correlation of different extents of excision of initial surgery with postoperative complications.

		Incomplete surgery	Complete surgery	*p* value
*n* (%)	69	17 (24.6)	52 (75.4)	—
Blood loss (ml)	144 ± 19	161 ± 36	138 ± 22	0.672
Length of hospital stay (days)	18 ± 1	21 ± 4	17 ± 1	0.206

Postoperative complications	*n* (%)			
None	34 (49.3)	8 (47.1)	26 (50.0)	0.833
Bile leakage	3 (4.3)	1 (5.9)	2 (3.8)	1.000
Choledocholithiasis	14 (20.3)	4 (23.5)	10 (19.2)	0.734
Cholangitis	30 (43.5)	8 (47.1)	22 (42.3)	0.732
Carcinogenesis	2 (2.9)	1 (5.9)	1 (1.9)	0.435
Surgical re-excision	12 (17.4)	6 (35.3)	6 (11.5)	0.025

**Table 7:** Statistical analysis of the correlation between postoperative outcomes and the potential risk factors.

Factors/*p* valve	BL > 150 mL	HS > 18 days	Carcinogenesis	Bile leakage	Choledocholithiasis	Cholangitis	*P*C	SR
Age at diagnosis (years)	0.400	0.303	0.002	0.002	0.376	0.524	0.592	0.247
Gender (female)	0.470	0.553	0.457	0.562	0.813	0.516	0.305	1.000
CCC type	0.384	0.296	0.967	0.996	0.873	0.292	0.343	0.937
Length of Y (cm)	0.249	0.429	0.425	0.885	0.151	0.735	0.643	0.424
Diameter of the CIA	0.092	0.343	0.097	0.987	0.040	0.002	0.000	0.387
Operative approaches	0.088	0.889	0.823	0.010	0.644	0.175	0.169	0.649

BL: blood loss, HS: hospital stay, PC: postoperative complication, SR: surgical re-excision, CCC: congenital choledochal cyst, CIA: cholangio-intestinal anastomosis, operative approaches include open, laparoscopic, and laparoscopic converted to open.

## Data Availability

The data used to support the findings of this study can be obtained from the corresponding author upon reasonable request.

## Abbreviation

CC: The choledochal cyst (CC)
MRCP: Magnetic resonance cholangiopancreatography
CIA: Cholangio-intestinal anastomosis
IQR: Interquartile range
PTCD: Percutaneous transhepatic cholangial drainage
ENBD: Endoscopic nasobiliary drainage
APBJ: Abnormal pancreaticobiliary junction.
